# Shared meditation involving cancer patients, health professionals and third persons is relevant and improves well-being: IMPLIC pilot study

**DOI:** 10.1186/s12906-022-03599-w

**Published:** 2022-05-18

**Authors:** Virginie Prevost, Sophie Lefevre-Arbogast, Alexandra Leconte, Claire Delorme, Sandrine Benoit, Titi Tran, Bénédicte Clarisse

**Affiliations:** 1 Normandie University, UNICAEN, INSERM U1086, ANTICIPE, 14000 Caen, France; 2 Centre François Baclesse, 14000 Caen, France; 3 National Clinical Research Platform for Quality of Life in Oncology, 162 rue Gabriel Péri, 94250 Gentilly, France

**Keywords:** Mindfulness, Pilot study, Cancer patients, Well-being, Health professionals

## Abstract

**Background:**

Alleviating suffering and improving quality of life are universally shared goals. In this context, we implemented a pilot study to assess the feasibility and acceptability of a mindfulness intervention in the form of meditation involving together cancer patients, health professionals, and third persons.

**Methods:**

Two groups of 15 participants equally composed of patients, health professionals and third persons were constituted. A dedicated programme on mindfulness and compassion was constructed, including 12 weekly sessions of 1.5 h and a half-day retreat. Adherence and satisfaction with the programme were evaluated. All participants completed questionnaires on perceived stress, quality of life, mindfulness, empathy, and self-efficacy. Burnout was assessed in health professionals.

**Results:**

Shared meditation was feasible as 70% of participants attended ≥ 80% of the 13 meditation sessions. Satisfaction with the programme was high (median satisfaction score: 9.1 out of 10) and all participants expressed positive attitudes towards shared meditation and a benefit on their global quality of life. Participants reported significant improvement in stress (*p* < 0.001), global quality of life (*p* = 0.004), self-efficacy (*p* < 0.001), and mindfulness skills (*p* < 0.001) from baseline to post-programme.

**Conclusions:**

This study demonstrated the feasibility of a shared dedicated meditation programme in terms of participation and acceptability of participants. The measured benefits observed among participants furthermore justify the interest of a subsequent randomized study aiming to demonstrate the potential added value of shared meditation by promoting bridge-building between cancer patients, health professionals and others.

**Trial Registration:**

ClinicalTrials.gov. NCT04410185. Registered on June 1, 2020.

**Supplementary Information:**

The online version contains supplementary material available at 10.1186/s12906-022-03599-w.

## Background

Cancer patients experience a significant symptom burden that often impacts their quality of life [1]. Pain is one of the most common symptoms and is prevalent in 55% of patients [2]. Cancer and its treatments may cause depression and anxiety in up to 20% and 10% of patients respectively, greatly exceeding the rate reported in the general population [3]. Medications are of limited efficacy and may induce side-effects. Thus, non-pharmacological approaches could also be used to help patients better manage their pain and other related symptoms.

In parallel, health professionals are particularly exposed to stress, anxiety, and depression due to the stressful nature of their work and are at high risk of burnout. This is especially the case in oncology where health professionals daily face life and death decisions [4, 5]. In addition, competent professionals are known to be committed not only to their patients’ well-being but also to their own [6], since burnout may have serious negative consequences for physicians, for patient outcomes as well as for the organizational coherence of the health care system [7].

Among the complementary therapies, mindfulness meditation is a mind–body intervention designed to alleviate suffering and stress-related symptoms and to promote well-being. Adapted from the age-old Buddhist meditation practices, mindfulness was first used by Kabat-Zinn in medical departments and society in the 1970s [8]. Kabat-Zinn defined mindfulness as non-judgmental attention to experiences of the present moment, including thoughts, emotions, and bodily sensations [8]. In particular, mindfulness-based stress reduction (MBSR) was initially developed for chronic pain patients [9]. Since then, it has been proven effective and is recommended in alleviating not only depression and anxiety in healthy people [10], but also in combatting the psychological distress associated with various types of diseases, including cancer.

An analysis of recent systematic reviews demonstrated the efficacy of mindfulness-based intervention in reducing combined measures of psychological distress and several other psychological and physical symptoms including anxiety, depression, fear of cancer recurrence, fatigue, sleep disturbances, and pain in cancer patients [11]. Another analysis found that all MBSR interventions in 13 reviewed studies had some positive effects on stress-related psychological or physiological outcomes, despite mixed results reported in four of these studies [12]. Zhang’s recent synthesis of 14 studies involving 1505 breast cancer patients highlighted the significant benefits of MBSR programmes on emotional well-being, anxiety, stress, and depression [13].

Regarding the impact of mindfulness on the suffering of health professionals, recent meta-analyses have shown that mindfulness reduces work distress [14, 15] and improves caregiver well-being [15]. MBSR interventions in health professionals were effective at improving mindfulness and self-compassion levels [16] as well as physicians’ well-being and performance [17]. On the other hand, very few studies have been published on the direct impact of mindfulness intervention on optimizing patient-health professional interactions, even if enhancing the well-being of health professionals is likely to improve the quality of care they provide [18–20]. Mindfulness has a positive impact on clinicians’ well-being by reducing stress and burnout and keeping them more present, which in turn affects the quality of communication they have with their patients, who in turn feel better understood [20].

As a response to suffering, shared meditation can offer the opportunity to build bridges and to reinforce mutual trust between the person being cared for (who wishes to play an active role in his or her disease) and the medical professional (who sometimes needs care), with whom a third party (‘non-patient’ and ‘non-care-giving’ subject) could be associated. In the context of this project, a third party is any person who is willing and motivated to participate. The third person therefore participates while sharing the suffering he or she may encounter in others but which he or she may also experience. In addition, the involvement of third persons in meditation workshops jointly to patients and health professionals might also help in destigmatizing the patient as a patient and the hospital as a place of care. Vulnerability, which is inherent in the human condition, not only affects those who experience illness [21]. Suffering is a shared experience, whether one is a medical professional, a patient, or a third person. “Anxiety may be allayed only when the health professionals and the cared-for find themselves on common ground, in the recognition that we all share joy and pain, life and death, and that the things that hurt and affect us also open us up to others and make us truly human” [22]. The recognition and sharing of this mutual vulnerability could lead to the emergence of creative, collaborative, and interactive health care.

Our focus is the putative added value of meditation in an open setting that associates cancer patients (target population), health professionals but also independent third persons in order to break out of the context of “illness” [23]. Indeed, experiments are being conducted at the Stress Clinic created in Massachusetts by Kabat-Zinn, where patients and medical staff come together to meditate [24], aiming at building bridges between medical professionals and patients. Yet could we not go one step further? We assume opening this approach to people who are neither health professionals nor cancer patients makes it possible to go beyond the context of the disease. By generating collective benefit in all three populations, meditating together might promote a sense of openness to each other and the feeling of sharing each other’s existence. The research hypothesis is that beyond the individual benefit meditating together may bring to participants, particularly in the daily management of stress, it may provide additional benefit in terms of well-being and strengthened links between patients, health professionals and third persons. Although supportive care has previously been offered occasionally to medical staff and patients in joint workshops in a clinical context but without research evaluating it, the hypothesis that additional benefit could be obtained by conducting the experience outside the context of the hospital and the illness has not yet been explored.

The IMPLIC pilot study herein reported is the first step in a comprehensive project aiming to evaluate the added value of "meditating together". As an initial step, this study was conducted in order (i) to validate the feasibility and acceptability of such a “shared meditation” with participants, and (ii) to evaluate the effects of meditation on improving well-being.

## Methods

### Clinical trial design

This was a longitudinal single-centre pilot study consisting of a non-randomized experimental pre/post intervention survey with minimal risks and constraints. The intervention under consideration was the delivery of a meditation programme in an open setting, involving simultaneously patients, health professionals, and third persons [23].

### Study participants

Concerning the recruitment, participants were informed about the study through posters displayed in the cancer centre and elsewhere, followed up by phone calls in order to check their eligibility. Patients were also recruited through posters displayed in the hospital’s meeting and information area. For the staff (including health professionals and third persons) from the François Baclesse Comprehensive Cancer Centre (Caen, France), hierarchical approval was obtained before validation of their inclusion in order to free the staff for the planned sessions. Importantly, the hospital’s director allowed staff to attend the workshops during their working hours and validated the inclusion of voluntary workers to ensure continuity of care and service.

Eligibility criteria are described in Table 1. The main parameter for inclusion was curiosity about meditation and motivation to be part of a study on it. Those recruited had to have no current or previous experience of regular or intensive meditation or comparable practice [25].

**Table 1:** Eligibility criteria

**INCLUSION CRITERIA**
** For all participants (patients, health professionals and third persons)**
- Participants aged 18 or over
- Curious and motivated to participate in programme
- Participant with no current or previous experience of regular or intensive practice of meditation or
- comparable practice. Practice considered regular and / or intensive if:
• it occurs more than one day per week for more than 6 consecutive months over the last 10 years,
• and/or in case of more than 5 consecutive days of intensive practice (internship or retirement) in the last 10 years,
• and/or more than 25 consecutive days of (cumulative) retirement over the last 10 years
- Participant available to follow full meditation programme (3-month period)
- Participant agreeing to participate by signing study information note
- Participant able to understand, speak and read French
- Participant able to use digital media and having internet connection
** Patient-specific criteria (target population)**
- Patients with cancer
- State of health compatible with study meditation programme
- Patient affiliated to a French social insurance system
** Health-professionals-specific criteria**
- All medical and/or paramedical health professionals of sponsor centre in contact with patients (doctors, nurses, nurse assistants, radiotherapy/radiology operatives…)
** Criteria specific to third persons**
- Any voluntary person not belonging to the two categories above
**NON-INCLUSION CRITERIA**
** For all participants**
- Participant with significant vulnerability factors: dependence on alcohol and drugs, severe depression, severe social anxiety, recent mourning etc.)
- Participant unable to participate for geographic, social or psychopathological reasons
- Participant deprived of liberty or under guardianship
** For patients**
- Very advanced stage of cancer disease with life-threatening symptoms
- Very advanced stage of cancer disease with life-threatening symptoms
** For third persons**
- Health professional, regardless of their place of practice

Cancer patients and health professionals were recruited at François Baclesse Comprehensive Cancer Centre. The health status of cancer patients had to allow them to attend the sessions. For medical and/or paramedical health professionals to be eligible, they had to be involved in the management of cancer patients (doctors, nurses, nursing assistants, radiotherapy/radiology operatives). As for third persons, any person not belonging to the above-mentioned categories was eligible, including people from outside the centre.

The eligibility of subjects presenting significant vulnerability such as very advanced cancer stage with life-threatening consequences, alcohol or drug dependence, severe depression, severe social anxiety, recent bereavement, etc. was assessed on a case-by-case basis. In this regard, the participant’s degree of commitment to the project was a determining criterion.

### Ethical approval

The IMPLIC trial was approved by the local ethics committee (Ref. CNRIPH: 20.03.20.55708, Comité de protection des personnes: Comity of person protection, Est II, France). This trial is registered as ID RCB 2020-A00485-34, ClinicalTrials identifier: NCT04410185, registered on 29/05/2020). All participants gave their written informed consent to the study before any study-related assessment. They could withdraw their consent at any time. The study was conducted under Good Clinical Practice Guidelines and the ethical principles of the Declaration of Helsinki [26].

### Mindfulness intervention

An expert in meditation was responsible for designing the programme and setting up the workshops. As a qualified instructor of MBSR programmes and member of the Medit-Ageing Research Group, she has taught meditation in the European Silver Santé Study project piloted by INSERM in Caen, France. This project sought to identify the factors of well-being and mental health in seniors [25]. Within that study, she developed a programme based on mindfulness and empathy that was adapted to our hypothesis concerning the benefits of "meditating together". In addition to the qualities of attentiveness and non-judgment, the programme also focused on peace of mind, caring and compassion.

**Fig. 1:**
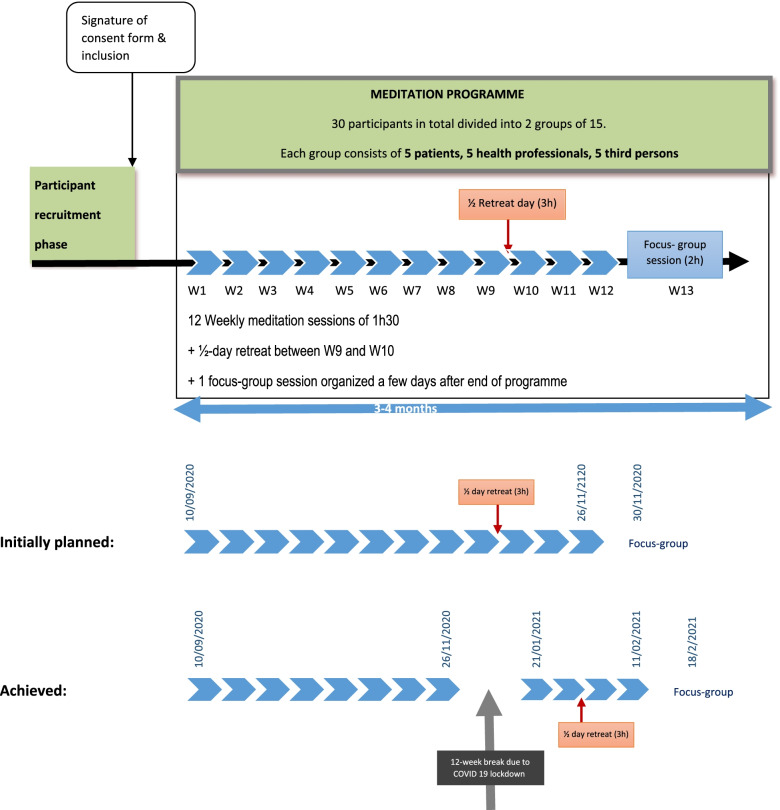
Study design

Regarding the organization of the meditation workshops, the reference protocol for stress reduction is the MBSR protocol [27]. This 8-week programme includes typically 8 weekly 2.5-h meditation sessions and an 8-h retreat day. As our target population is cancer patients, we thought sessions lasting 2.5 h plus a one-day retreat might be too longer for some patients, so we opted for more but shorter sessions over a period of 3 months, i.e. 12 weekly sessions of 1.5 h of meditation and a half-day retreat of 3 h (Fig. 1) [23]. The programme was administered to two groups of participants equally distributed across patients, health professionals, and third persons.

Each weekly session included a time of welcome and a tour de table to check everyone’s internal weather (around 10 min), followed by the presentation of the theme covered for the session (15 min), a practice of meditation on this theme (10 min), a time sharing on the feedback, a second period of practice and mindful movements (10 min), a 15-min break, a question-and-answer time addressed the questioning points raised by the session, and, lastly refocusing and presentation of daily practice exercises to work at home (5 min). The themes addressed during these meditation sessions dealt with mindfulness, and explored more especially breathing, sensations, sounds, body scan, and impermanence… Empathy was approached through themes such as interdependence, compassion, wisdom… The half-day retreat occurred in silence. It aimed to allow participants exploring meditation in more depth, over a longer period, in order to promote the integration of the meditation techniques discussed during the sessions. Its goal was to allow participants to reconnect with themselves and include their practice in a context conducive to direct experience. It was an opportunity to propose, in addition to the themes of previous sessions, sharing meal time (meditation on taste), a meditative walk outside, and a debriefing to close this experience.

As part of the programme, participants were required to complete daily practice exercises and meditation sessions at home. To guide them, written and audio materials specially designed for this purpose by the teacher were also provided. Finally, individual follow-up was offered, if necessary, by phone, video call or face-to-face discussion to resolve any problems raised during the sessions that could not be managed within the group. The meditation sessions and the deepening session (retreat) took place outside the hospital, allowing for meetings and exchanges in a neutral, friendly and convivial environment (*Pôle des Formations et de Recherche en Santé* of Caen University), a pleasant setting conducive to exchange, close to the François Baclesse Comprehensive Cancer Centre.

### Study endpoints

#### Primary outcome measure

The main objective was to evaluate the participants’ adherence to the 13-session meditation programme. In drug treatment studies, good adherence is usually defined as an 80% or more ratio of doses taken out of prescribed doses [28]. Similarly, in our study, participants were considered adherent when they attended at least 80% of the 13 sessions of the whole meditation programme (12 weekly sessions and one half-day retreat).

#### Secondary Outcome measures

As secondary objectives, the acceptability to the meditation programme was evaluated at the end of the programme through the satisfaction of participants with regard to the intervention, their feeling about meditating together and their perception of change in their overall health and quality of life.

##### Participant satisfaction

The overall degree of satisfaction of participants was assessed on a visual scale ranging from 0 (*not satisfied at all*) to 10 (*totally satisfied*). Then, a number of item were specifically rated on a 5-point Likert scale including the schedule of the programme, its content and the pedagogical techniques used, the quality of the relationship with the trainer and the exchange with/among the participants.

##### Putative benefit of "Meditating together"

We developed questionnaires specifically for this study to evaluate the putative benefit of "meditating together". The global opinion of participants was assessed on a visual scale ranging from -5 (*very harmful*) to + 5 (*very beneficial*). To allow us to refine the overall response, we also used Likert scales specific to all three types of participant to assess their feelings about meditating together in terms of discomfort, value and facilitation of communication and compassion between patients, health professionals and third persons.

##### Perception of change in overall health and quality of life

Participants’ perception of change in their overall health and quality of life was measured at the end of the programme using a visual scale rated from -5 (*significant deterioration*) to + 5 (*considerable improvement*).

#### Exploratory outcome measures

As exploratory goals, the study also aimed to assess the putative effects of meditation on stress and quality of life, on feelings of personal effectiveness, on the degree of burnout (in health professionals only) and on the development of mindfulness and empathy. These exploratory outcomes were assessed pre- and post-intervention (Fig. 1).

##### Quality of life, stress, and life management in participants

In all participants, quality of life was globally assessed using a visual analogue scale [29], which provided a continuous value between 0 (*very bad quality of life*) and 10 (*excellent quality of life*). The perception of stress was measured using the 10-item Perceived Stressed Scale (PSS) questionnaire [30], which is validated in French [31]. Scores on the PSS range from 0 to 40, with higher scores indicating a higher level of stress [30].

The 10-item Generalized Self Efficacy Scale (GSE [32]) validated in French [33] and commonly used in positive psychology was used to assess the ability and motivation to cope with difficult situations.

In the health professionals only, burnout was assessed with the Maslach Burnout Inventory (MBI, [34]) translated in French [35]. This scale explores subjective experience and feelings linked to the emotional and affective state of an individual at work through three dimensions: emotional exhaustion (EE), depersonalization (DP) which indicates unfeeling and impersonal responses toward patients, and personal accomplishment (PA). A high score in EE and DP or a low score in PA indicate a high risk of burnout [36].

##### Qualities of mindfulness and empathy

To assess mindfulness, participants completed the short 14-item version of the Freiburg Mindfulness Inventory (FMI, [36–38]), which has been validated in French [39]. The FMI assesses the general tendency to be mindful in everyday life by exploring two sub-facets: presence and acceptance. It was developed for people without background knowledge in mindfulness as it is semantically independent from Buddhist or meditation discourse. It is therefore applicable to all population groups.

The interpersonal reactivity index (IRI) developed by Davis [40] and validated in French [41] was used to evaluate the spontaneous tendency to empathy. It is a 28-item questionnaire exploring four dimensions: perspective-taking, fantasising, empathic concern and personal distress. The Jefferson Scale of Empathy (JSE, [42]) is a widely used instrument that measures empathy in the context of health professions’ education and patient care. First developed for medical staff [43], it was then adapted for health students [42]. In the latter, the items were phrased in a neutral manner, which allowed us to administer the validated French version [44] to all participants. The total JSE score and the three dimensions of the JSE (perspective-taking, compassionate care, standing in patients’ shoes) were computed.

### Statistical analysis

For this pilot study aiming at examining the feasibility and acceptability of the intervention, we included 2 groups of 15 participants equally distributed among patients, health professionals and third persons. Characteristics of participants were described using number and proportion for categorical variables and median and range for continuous variables, and were compared across patients, health professionals and third persons using Kruskal–Wallis and Chi-square tests.

The proportion of adherent participants was estimated with its 95% confidence interval (95% CI) based on the principles of intention-to-treat analysis.

In secondary analyses, we described the global satisfaction of participants, the perceived benefit of "meditating together” and the perception of change in their quality of life (all evaluated at the end of the programme through visual scales) using median scores and interquartile ranges (IQR). We used radar chart to display the average satisfaction of participants on specific dimensions of satisfaction in the total sample and by subgroup (patient/health professional/third person). Feelings about meditating together were explored through Likert scale graphical visualization.

In exploratory analyses, median scores and interquartile ranges (IQR) of quality of life, stress, mindfulness and empathy were computed pre- and post-intervention in the total sample. We tested for the effect of the intervention among all participants using paired Wilcoxon tests comparing pre- and post-evaluations. Non-parametric tests were chosen because of the small sample size. Z-test statistic and effect size were provided in addition to *p*-values.

Only participants who completed one final evaluation were included in secondary and exploratory analyses.

All statistical analyses were carried out using R statistical software (4.1.0).

## Results

### Participant enrolment and flow

**Fig. 2:**
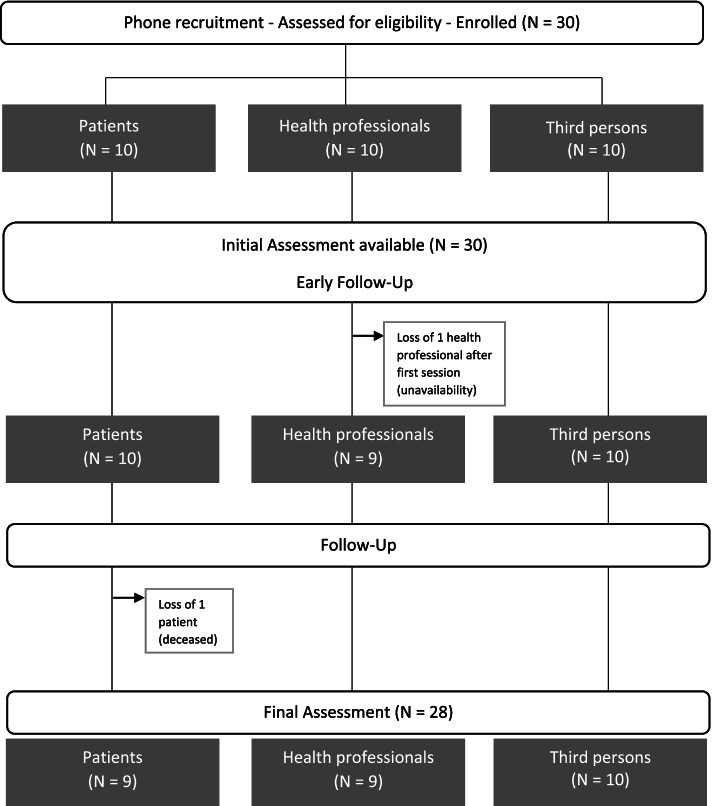
IMPLIC study flow chart

A total of 30 participants (10 patients, 10 health professionals, 10 third persons) were enrolled in the study (Fig. 2) and included in the main analyses on programme adherence. One caregiver (nursing assistant) who completed the initial assessment left the study after the first session due to lack of availability, and one patient deceased before the end of the programme. Thus 28 participants (9 patients, 9 health professionals, 10 third persons) completed the post-intervention questionnaires and were included in secondary and exploratory analyses.

### Participant characteristics

Characteristics of participants are described in Table 2.

**Table 2:** Sociodemographic characteristics of participants and meditation experience (*N* = 30)

	**Patients** (*N* = 10)	**Health professionals** (*N* = 10)	**Third persons** (*N* = 10)	***P* value**	**Total** (*N* = 30)
**Age,** median years (range)	60 (40–80)	46 (30–60)	57 (38–79)	0.017	54 (30–80)
**Sex, n (%)**				0.32	
Female	8 (80)	10 (100)	8 (80)		26 (87)
Male	2 (20)	0 (0)	2 (20)		4 (13)
**Last profession, n (%)**				0.079	
Executives and higher intellectual	4 (40)	5 (50)	7 (70)		16 (53)
profession	1 (10)	5 (50)	2 (20)		8 (27)
Employees	4 (40)	0 (0)	1 (10)		5 (17)
Intermediate occupation	1 (10)	0 (0)	0 (0)		1 (3)
Other persons not in employment					
**Health professionals’ profession, n (%)**					
Nursing assistants		1 (10)			
Doctor		3 (30)			
Nurse		1 (10)			
Radiotherapy/radiology operatives		3 (30)			
Health executive		2 (20)			
**Experience of meditation, n (%)**				0.32	
Naive	8 (80)	10 (100)	8 (80)		26 (87)
Sensitized	2 (20)	0 (0)	2 (20)		4 (13)

*P*-values are from Kruskal–Wallis test for age and Chi-square tests for other categorical variables

The 30 participants were mostly women (87%) and most were likely to have postgraduate education (53% executives/higher intellectual professions). Median age was 54 years, with health professionals being slightly younger than third persons and patients (median 46, 57 and 60 years old, respectively). Among the health professionals, the most represented professions were physicians and radiotherapy/radiology operatives (30% each). The third parties were found to have no connection with patients or health professionals. There was no difference in sociodemographic characteristics between the two groups of 15 participants who attended the meditation programme.

### Adherence to meditation programme

The overall adherence rate was 70% (95% CI = 51%-85%). Nine participants (30%) attended all 13 sessions. The mean number of attended sessions over all 13 programme sessions was 10.5 (SD = 2.7) and was similar in patients (10.5 ± 2.4), health professionals (10.4 ± 3.9), and third persons (10.7 ± 1.9). Sixty percent of subjects participated in at least 80% of the 12 weekly sessions, and 73% of participants, equally distributed among the three groups (7 patients, 7 health professionals, 8 third persons), attended the half-day retreat.

Reasons for non-attendance at the weekly sessions included medical reason (including COVID-19) and medical treatment (43%), participant’s unavailability or delay (28%) and participant’s preference (5%) or were unspecified (24%).

During session intervals, patients were asked to perform daily meditation exercises based on audio recordings provided by the instructor. Daily meditation exercises at home were partially and fully completed by 90% (8 patients, 9 health professionals and 10 third persons) and 6% (2 patients) of participants, respectively. During the programme, the participants performed exercises for 53 days on average (58 for patients, 55 for third persons and 46 for health professionals). The main reasons for non-completion of inter-session exercises mainly included lack of time (77%) and/or lack of motivation (40%), and/or to a lesser extent fatigue (17%), illness (7%) and forgetfulness (40%).

### Participants’ satisfaction, feelings about "meditating together" and perception of change in quality of life

**Fig. 3:**
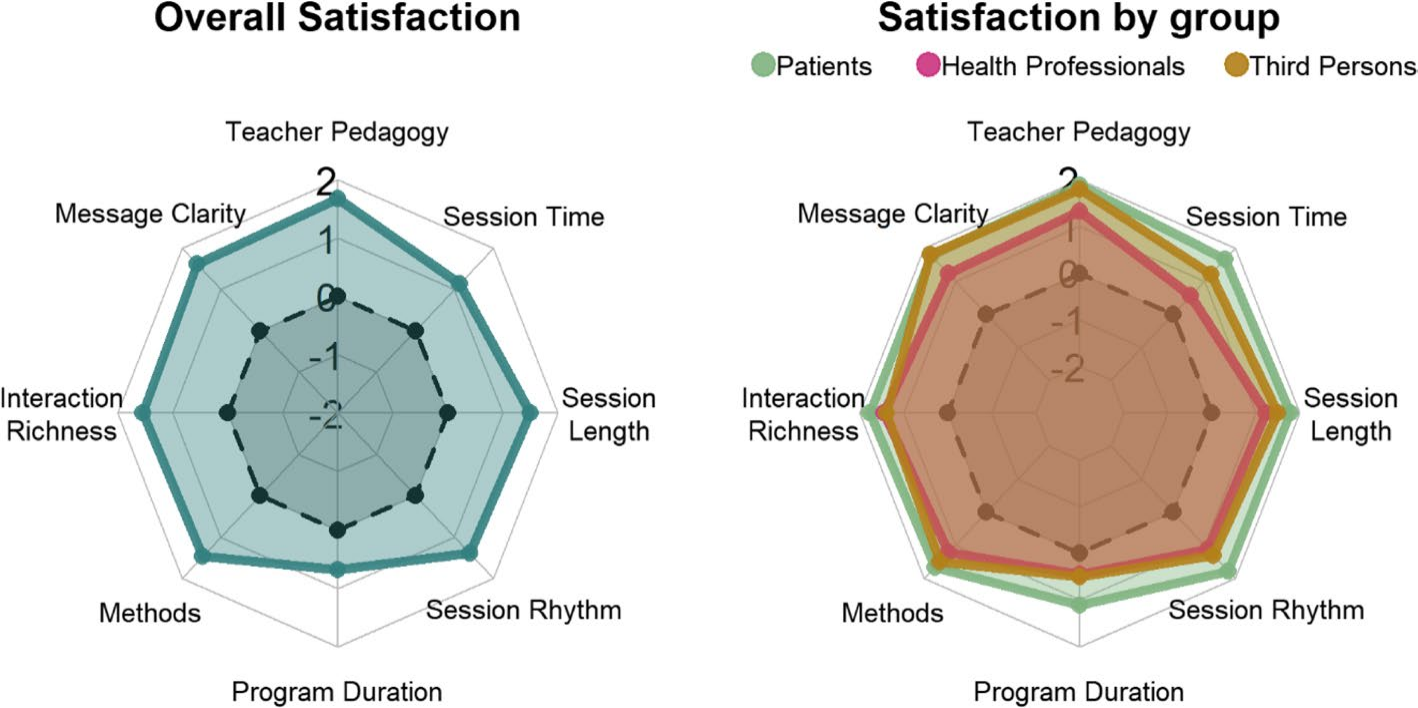
Participants’ satisfaction per component (*N* = 28) Each component of satisfaction was rated on a 5-point Likert scale from -2 (*very dissatisfied*) to + 2 (*very satisfied*). Radar charts display for each component the average satisfaction of participants in the total sample (left panel) and across the three groups (right panel). The zero line divides the scale from dissatisfaction to satisfaction. The closer the point to the centre of the axis, the larger the dissatisfaction; the closer the point to the end of the axis, the larger the satisfaction

Participants were asked about their level of satisfaction concerning the programme and its organization. The median degree of satisfaction with the programme was 9.1 (IQR = 8.6–9.4) on a scale from 0 (*not satisfied at all*) to 10 (*totally satisfied*). Only one participant from the third person group was not satisfied (score 3.9) and mentioned in an open-ended comment the lack of interaction with other participants. On average, positive satisfaction scores were reported by the participants on all components of the programme (Fig. 3, Overall Satisfaction). Specifically, most participants were *satisfied* or *very satisfied* with teacher’s pedagogy (96%), message clarity (96%), richness of interaction (97%), pedagogical methods (90%), session rhythm (93%), and session length (93%). Satisfaction was lower for session time (79%), and programme duration (68%). On average, satisfaction ratings were higher in patients and lower in health professionals on all components, especially session time and programme duration (Fig. 3, Satisfaction by group). Dissatisfied participants reported in the open-ended comment field that the programme was too short, but did not comment on the session time. All participants reported that the programme had changed their habits and/or behaviours, except two health professionals.

**Fig. 4:**
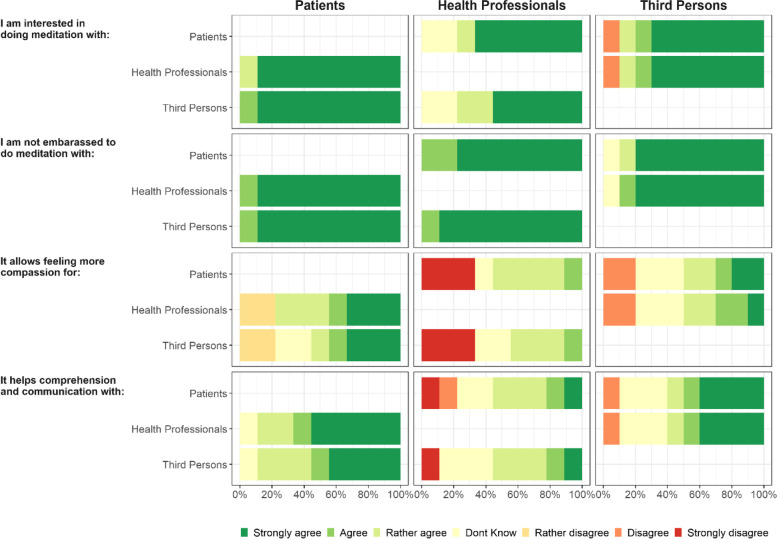
Participants’ feeling about meditating together (*N* = 28)

Regarding the participants’ global opinion of “meditating together”, they had a positive attitude towards shared meditation at the end of the program, with a median score of 3.7 (IQR = 3.0–4.4) on a scale ranging from -5 (*very harmful*) to 5 points (*very beneficial*). No participant felt it had been even minimally harmful. When we explored specific feelings about meditating together (Fig. 4), we observed that globally patients agreed on all features of meditating together, including the interest, comfort, improvement of compassion and communication/comprehension. As for health professionals and third persons, they also reported interest and comfort about meditating together. However, half of both groups agreed with its benefit on compassion and communication.

At the end of the programme, the median score (scale from -5 to + 5) of participants’ perception of change in their overall health and quality of life was 2.7 (IQR = 1.6–3.3, min = 0, max = 4.7). All participants reported improvement in their global quality of life, except one patient who reported no change.

### Effect of programme on stress, well-being and life management ( Table 3)

**Table 3:** Pre- and post-intervention exploratory outcome measures of participants (*N* = 28)

	**Scale Range**	**Baseline** **Median**	**Post-test** **Median**	**Z test statistic**	**Effect size** ^**a**^	***P*** ^**b**^
**Global Quality of life**	0–10	6.7	7.4	2.87	0.54	0.004
**Perceived Stress Scale (PSS)**	0-40^c^	21	15	3.48	0.66	< 0.001
**Total Score Mindfulness (FMI)**	14–56	35	38^d^	3.28	0.62	0.001
**Presence**	6–24	16	17^d^	2.26	0.43	0.024 < 0.001
**Acceptance**	8–32	20	22	3.42	0.65	
**Total Score Empathy (IRI)**						
**Perspective-Taking**	7–35	25	27^d^	0.89	0.17	0.37
**Fantasy**	7–35	23	21^d^	0.47	0.09	0.64
**Empathic Concern**	7–35	31	31^d^	0.51	0.10	0.61
**Personal Distress**	7–35	17	15^d^	0.85	0.16	0.40
**Total Score Empathy in Care (JSE)**	20–140	108^d^	104^d^	1.36	0.26	0.28
**Perspective-Taking**		57	54	1.70	0.32	0.090
**Compassionate Care**	10–70	46	43	1.54	0.29	0.12
**Standing in Patient’s shoes**	8–56 2–14	6.0^d^	8.0^d^	1.60	0.30	0.11
**Self-Efficacy Scale (GSE)**	10–40	30 ^d^	33	3.51	0.66	< 0.001
**Burnout (MBI,** Health professionals only, *N* = 9**)**						
**Emotional Exhaustion**	9-63^c^	33^d^	28	0.68	0.26	0.50^e^
**Depersonalization**	5-35^c^	12^d^	8	0.41	0.15	0.68^e^
**Personal accomplishment**	8–56	41^d^	51	1.44	0.54	0.15^e^

^a^ Effect sizes are computed as the ratio of the Z test statistic to the square root of the number of pairs and range from 0 to 1 with higher values indicating larger effect

^b^ *P*-values are from paired Wilcoxon test comparing pre- and post-test scores

^c^ High score indicates worse state

^d^ Indicates 1 missing questionnaire, except for MBI (two missing baseline evaluations)

^e^ *P*-value for pre-post comparison among health professionals only

The global quality of life score (visual analogue scale 0–10) improved after the programme from a median baseline score of 6.7 (IQR = 5.0–7.5) to a median score of 7.4 (IQR = 6.7–8.2) (*p* = 0.004). A significant reduction in perceived stress was observed in the global population following the programme, with a median score decreasing from 21 (IQR = 16–25) to 15 (IQR = 11–19) (*p* < 0.001). Stress reduction was particularly marked in health professionals, whose scores dropped by 10 points on average, compared to 5 and 2 points among patients and third persons, respectively.

Self-efficacy improved significantly in the global population with a median score increasing from 30 (IQR = 27–33) to 33 (IQR = 30–36) (*p* < 0.001).

Regarding burnout assessed in 7 health professionals at baseline (two missing questionnaires), scores indicated high levels of emotional exhaustion and depersonalization (median scores of 33 and 12, respectively) but also high personal accomplishment (median of 41). There was no significant change in MBI scores following the programme (all p for pre-post comparison ≥ 0.15), although all scores improved and large effect size was observed for personal accomplishment (Z-test statistic = 1.44; effect size = 0.54).

### Effect of programme on mindfulness and empathy ( Table 3)

Mindfulness quality scores were significantly improved after the programme. FMI scores showed a significant increase in all participants between baseline and post-intervention: total median score (35 [31–40] to 38 [36–45], *p* = 0.001) and its two components of presence (16 [14–19] to 17 [16–21], *p* = 0.024) and acceptance (20 [17–23] to 22 [20–23], *p* < 0.001). Acceptance, which represents self-acceptance or non-judgmental acceptance of experience, was found to be particularly improved in health professionals (median score increased by 5 points, results not shown).

Concerning multidimensional empathy, no statistical difference was observed following the programme when comparing empathy scores assessed by IRI in all participants, with effect size being rather small whatever the sub-dimension (all effect sizes ≤ 0.17). The participants’ perception of the importance of empathy in care assessed with the JSE was not modified by the intervention either on total median scores or on those of the three sub-scales. Effects size for JSE scores were moderate ranging from 0.26 for the total score to 0.32 for the perspective-taking dimension.

## Discussion

The prevalence of anxiety and depression among people with cancer is often higher compared to that of the general population and is therefore a clinical and research priority [45]. In this respect, the benefits of mindfulness are increasingly being observed in cancer management [46]. The findings of our pilot study highlight the feasibility and acceptability of organising a meditation programme associating cancer patients, oncology health professionals and third persons. Adherence was relatively high, as 70% of participants followed at least 80% of the programme, a figure similar in the three groups. We also assessed the potentially harmful effects of the intervention, since worsening of participants’ overall state has been observed by others [47, 48]. However, none of our participants reported any adverse effects or worsening of their state after the intervention.

Although the design of this study did not allow us to demonstrate clearly whether shared meditation has a measurable added value, its feasibility was clear. Our meditation programme differed from standard MBSR programmes since it was specifically designed for patients in terms of content and modalities and was highly appreciated by all participants. Patients were particularly enthusiastic about its delivery and the fact that both health professionals and third persons were prepared to meditate together with them. The benefit of the presence of third parties, contributing to the development of a sense of common humanity, beyond care, will be further analysed through qualitative focus group analysis. The only shortcoming identified with the programme was its shortness, an opinion expressed by the participants in the free comment fields.

The findings also suggested an improvement on several dimensions of well-being across the intervention. However, this pilot feasibility study was not designed to prove the benefits of meditating in an open setting (e.g., no control group, small sample size), and in the absence of a control group, we cannot exclude that changes in outcomes may be due to other processes over time. These findings are preliminary and require further evaluation through a larger and methodologically robust study, especially since data on shared meditation in oncology settings are still sparse.

Nevertheless, participants reported significant improvement in stress, quality of life, self-efficacy and mindfulness qualities from baseline to post-programme. Of note, these indicators were found to be similar at baseline for all categories of participants, including quality of life parameters, even for cancer patients. Similarly, recent meta-analyses reported better quality of life and lower stress following mindfulness-based interventions in the global population [10] and in cancer patients [11, 49]. Perceived stress is defined as the extent to which persons perceive that their demands exceed their ability to cope [30]. The simultaneous reduction in stress and improvement in the feeling of self-efficacy that we observed may result from the mindfulness intervention that allows individuals to better perceive their own point of view in a situation and thus to better focus on their objectives. Various tools can be used to assess the qualities of mindfulness [50]. Our programme promoted the development of meditation skills as assessed by the FMI, even though multifaceted instruments might assess the breadth of the mindfulness construct more fully and therefore better capture the skills that specifically change with mindfulness training [51].

Mindfulness did not have a significant effect on empathy in our participants. Several hypotheses have been put forward on how the practice of mindfulness can promote an increase in empathy [52]. These include self-awareness, non-reacting, and non-judging. Becoming aware of the present moment in time may make individuals more aware of another person’s experience and what they are feeling. Although there is some empirical consensus whether mindfulness increases empathy efficiently [52], how to demonstrate it remains a matter of debate. The amount of mindfulness practice is very likely one factor [53], as are the tools used to assess it. In this study, we used the widely used IRI, although its efficacy is still discussed [54]. Another possible bias is that the items of the IRI used to measure affective empathy require individuals to resort to cognitive empathy in situations created by the item before making emotional responses [55]. In addition, the very relevance of the self-reporting of empathy is an issue at the conceptual level. However, while there is a rationale for the link between meditation practice and empathy, and although this dimension was built into the programme, its rollout during the COVID-19 pandemic may also have impacted the fostering of empathy. While the main aim of the study was to promote communication between patients and oncology health professionals, the results of the JSE did not reveal any effect of the programme on empathy. On the other hand, verbatim responses collected in the satisfaction questionnaires showed that meditation did have an effect on patient-health professional interaction, as exemplified by remarks like “*it creates 'commonality’ between us and humanises relationships*”. Meditation could thus have a highly positive effect, as other authors found [18]. Mindfulness might help health professionals not only to heal their patients but also to heal themselves [56], in a context of high professional pressure. By becoming compassionately aware of the pain of others without getting lost in it, harmful effects such as empathy fatigue and empathic distress can be reduced [57]. Mindfulness-based interventions have already shown their value in attenuating the symptoms of burnout [58]. Our findings suggest a possible reduction in burnout following the programme regarding the three dimensions addressed, although it is not significant given the small number of carers.

Concerning the optimisation avenues for the construction of a future randomised study to evaluate the potential added value of shared meditation, increasing the patient ratio to half of the participants with 25% of health professionals and 25% of third persons would strengthen our target population. It would be also consistent with future rollout of the programme in hospitals, in view of the high turnover of patients. Self-compassion should also be assessed since it can predict change in most other outcomes and can therefore be a critical factor in the effectiveness of a programme [59]. As our mental and emotional states are interconnected, patients are known to resonate with their caregiver’s more positive mental state when the latter express self-compassion regarding the difficulty of caring for patients [58]. Finally, follow-up sessions would allow the effects of meditation to be extended, sustained and re-evaluated over time. Another avenue of investigation is the complementary qualitative analysis of focus groups conducted at the end of this pilot study to establish the key determinants of the effects of shared meditation especially for patients, leading to an optimally designed randomized study.

This pilot study has some limitations. First, this “self-selected” population based on voluntary participation mainly comprised women from a privileged socio-professional background, so the findings cannot be generalised. Second, although the hospital director allowed the staff to participate during their working hours, some members of staff were unable to do so for organisational reasons because their absence would have impacted the smooth running of their department. This was particularly the case for nurses or nursing assistants. This underlines the need to optimise the hospital’s workflow to allow all staff to follow this kind of programme. Third, the sample was small, which limits the power of the statistical analyses on exploratory outcomes.

A final and important limitation is that the programme began in September 2020 during the COVID-19 pandemic, i.e. an unfavourable context. Notably, the second nationwide lockdown at the end of 2020 required a 12-week break in the meditation programme after the eighth session. Yet although this might have had an impact on adherence, it remained satisfactory. Notably, to maintain contact with the participants during this period, the meditation instructor encouraged them to continue the practice at home as much as possible using the audio material already provided, and offered them an additional supportive audio session with the opportunity to contact her if needed. In addition, social distancing measures and mask-wearing were not conducive to exchange between the participants, and it was sometimes difficult for patients to attend the sessions. Indeed, one-off catch-up sessions with individual participants had to be organised by videoconference. The context of the pandemic may have affected the evaluation of the effects of shared meditation on various parameters of well-being.

## Conclusions

Despite its limitations, this pilot study shows the feasibility and acceptability of a programme in which cancer patients, health professionals in oncology and third persons meditate together. The programme had a positive effect in reducing stress and improving well-being, self-efficacy, and mindfulness skills in the three categories of participants. The study lays the foundations for a future randomized study to demonstrate the putative added value of shared meditation. It offers the perspective of improving cooperation between patients and health professionals, this engendering mutual benefit. Mindfulness could thus constitute a powerful tool to enhance well-being and to alleviate suffering, with the understanding that suffering is part of shared human experience.

## Supplementary Information

**Additional file 1.** STROBE Statement—checklist of items that should be included in reports of observational studies.

## Data Availability

Available from the corresponding author upon reasonable request.

## Abbreviations

DP: Depersonalization
EE: Emotional Exhaustion
FMI: Freiburg Mindfulness Inventory
GSE: Generalized Efficacy Scale
IQR: Interquartile ranges
IRI: Interpersonal Reactivity Index
JSE: Jefferson Scale of Empathy
MBI: Maslach Burnout Inventory
MBSR: Mindfulness-Based Stress Reduction
PA: Personal Accomplishment
PSS: Perceived Stress Scale
